# Measuring Technique for Meniscal Extrusion Using Ultrasound in the Setting of Posterior Medial Meniscal Root Tears

**DOI:** 10.1016/j.eats.2024.102916

**Published:** 2024-01-30

**Authors:** Daniel Farivar, Tomás A. Pascual, Mario Hevesi, Jorge Chahla

**Affiliations:** a Department of Orthopaedic Surgery, Division of Sports Medicine, Midwest Orthopaedics at Rush, Rush University Medical Center, Chicago, Illinois, U.S.A.; b Department of Radiology, HIMAN Barrio Norte, Buenos Aires, Argentina; c Department of Orthopedic Surgery, Mayo Clinic, Rochester, Minnesota, U.S.A.

## Abstract

Meniscal extrusion (ME) is a valuable, noninvasive diagnostic tool for meniscus pathology. In addition, ultrasound can allow for instant results in the office. However, ME is a fragile metric in the degree of millimeters. The inconsistent techniques used by different authors in the literature and the additional operator variability that ultrasound technology introduces make it important a systematic approach is used. The purpose of this study is to propose a reproducible technique. The current authors recommend having the patient in (1) 30° of knee flexion while weight-bearing, (2) capturing the image at the posterior border of the medial collateral ligament, (3) using a reference line parallel to the articular margin of the tibia, and (4) measuring ME at the coronal midpoint of the outermost edge of the meniscus.

Posterior medial meniscal root tears (PMMRTs) are debilitating injuries that disrupt the load-distributing function of the medial meniscus.^1^ As a result, the knee experiences pathologically increased loads and patients may develop early-onset medial compartment osteoarthritis.^2^ Due to the loss of a major attachment site during PMMRTs, the medial meniscus may displace off the tibial plateau, resulting in meniscal extrusion (ME).^3^ Previous studies have shown that ME is an independent predictor for developing subchondral bone lesions and subsequent tibiofemoral cartilage degeneration.^4^^,^^5^ To date, numerous cut-offs have been used to indicate pathologic ME. Currently, ME greater than 3 mm has generally been accepted as indicating disease, but there are other studies proposing cut-offs of 2 mm, 2.5 mm, and even 4 mm of ME.^6^, ^7^, ^8^, ^9^, ^10^

Although ME can provide a noninvasive way for an orthopaedic surgeon to both qualitatively and quantitatively assess the meniscus, the details of its measurement technique have not been discussed in a granular manner in the orthopaedic literature. A recent meta-analysis demonstrated that ME pooled from all patients with PMMRTs was 3.2 ± 2.0 mm—a wide range.^11^ In addition, they also found that authors measured ME at various landmarks, including the medial collateral ligament in 38% of studies, the midpoint of the anterior−posterior length of the medial meniscus in 23% of studies, the middle of the medial femoral condyle in 19% of studies, and more. Given its low absolute measured values in the order of millimeters, ME is a fragile metric and requires a systematic and reproducible measurement technique so quantitative values can be meaningful. This is likely the only way ME cut-offs can reliably be developed and used. Therefore, the purpose of this study was to propose a step-by-step approach for measuring ME using ultrasound.

## Surgical Technique (With Video Illustration)

The goal when measuring ME with ultrasound is to first achieve a clear image of the relevant anatomy using a consistent approach for all patients. Although any functioning ultrasound machine will be appropriate, we used the My Lab X8 Ultrasound Machine (Esaote, Genoa, Italy) with the L4-15 High Frequency Probe placed in the “MSK” preset. A line of conductive gel should then be applied on the covered probe surface and can be reapplied throughout the procedure as needed.

**Fig 1:**
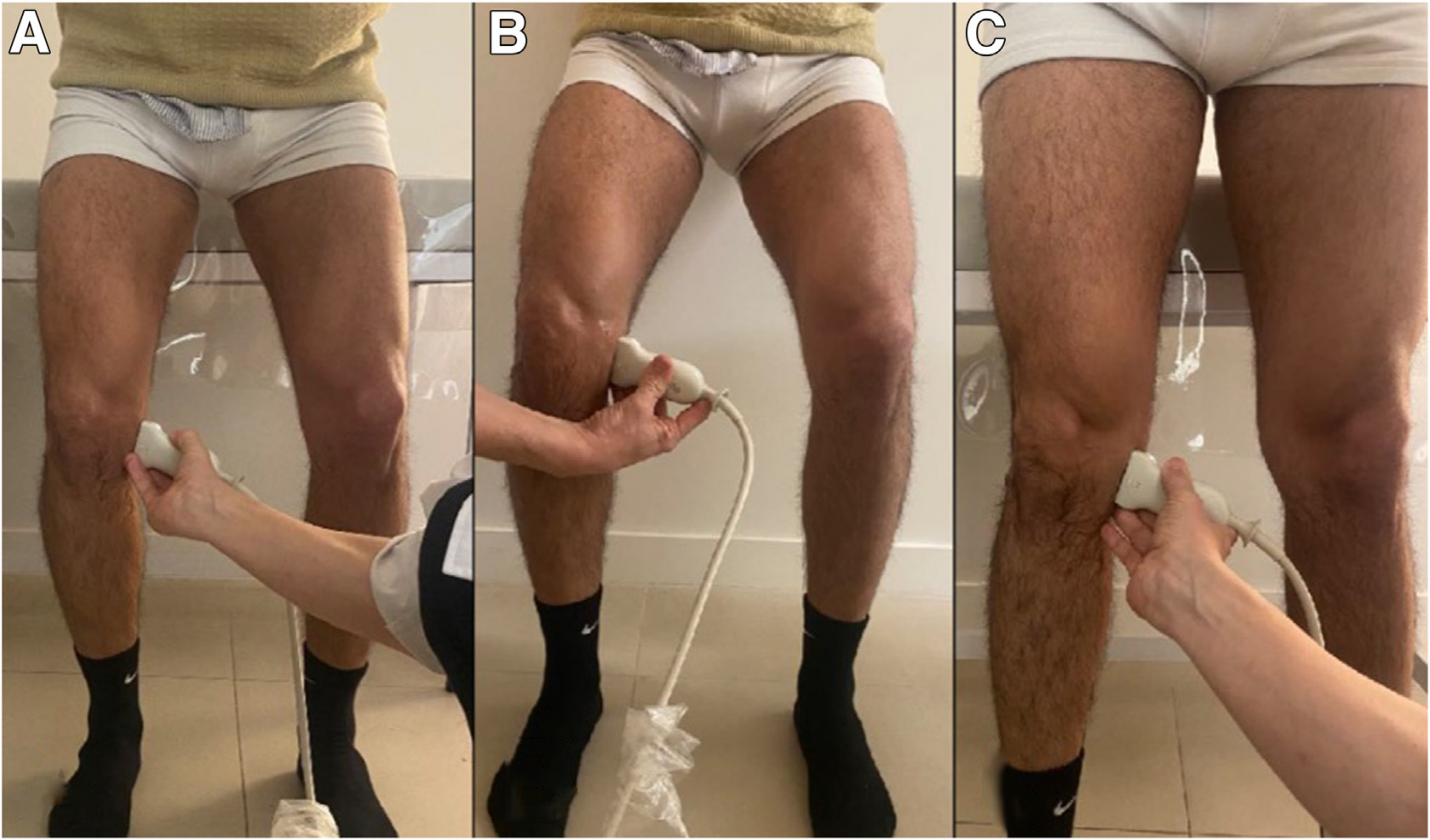
When measuring ME, the patient should bend the knees to about 30° of flexion while bearing weight (A). If the patient cannot tolerate this position, he can use a nearby wall for support (B). Lastly, ME can also be measured in full extension (C). (ME, meniscal extrusion)

**Fig 2:**
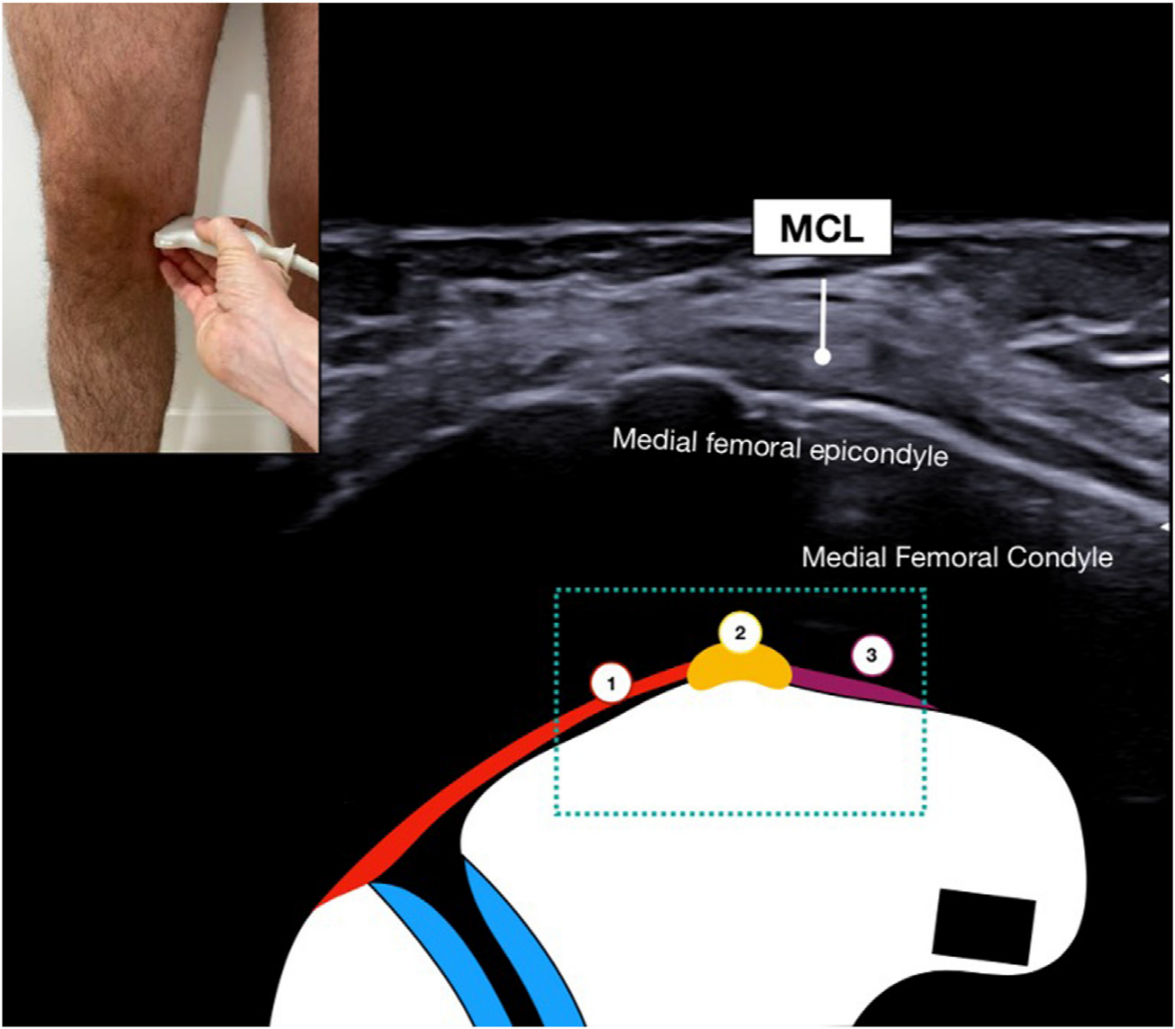
When measuring medial ME from an ultrasound image, the first step is identifying the MCL. Begin by placing the probe over the medial femoral epicondyle in the short-axis plane. The MPFL can be found anterior to the MCL, whereas the POL can be found posterior to the MCL. (MCL, medial collateral ligament; ME, meniscal extrusion; MPFL, medial patellofemoral ligament; POL, posterior oblique ligament.)

**Fig 3:**
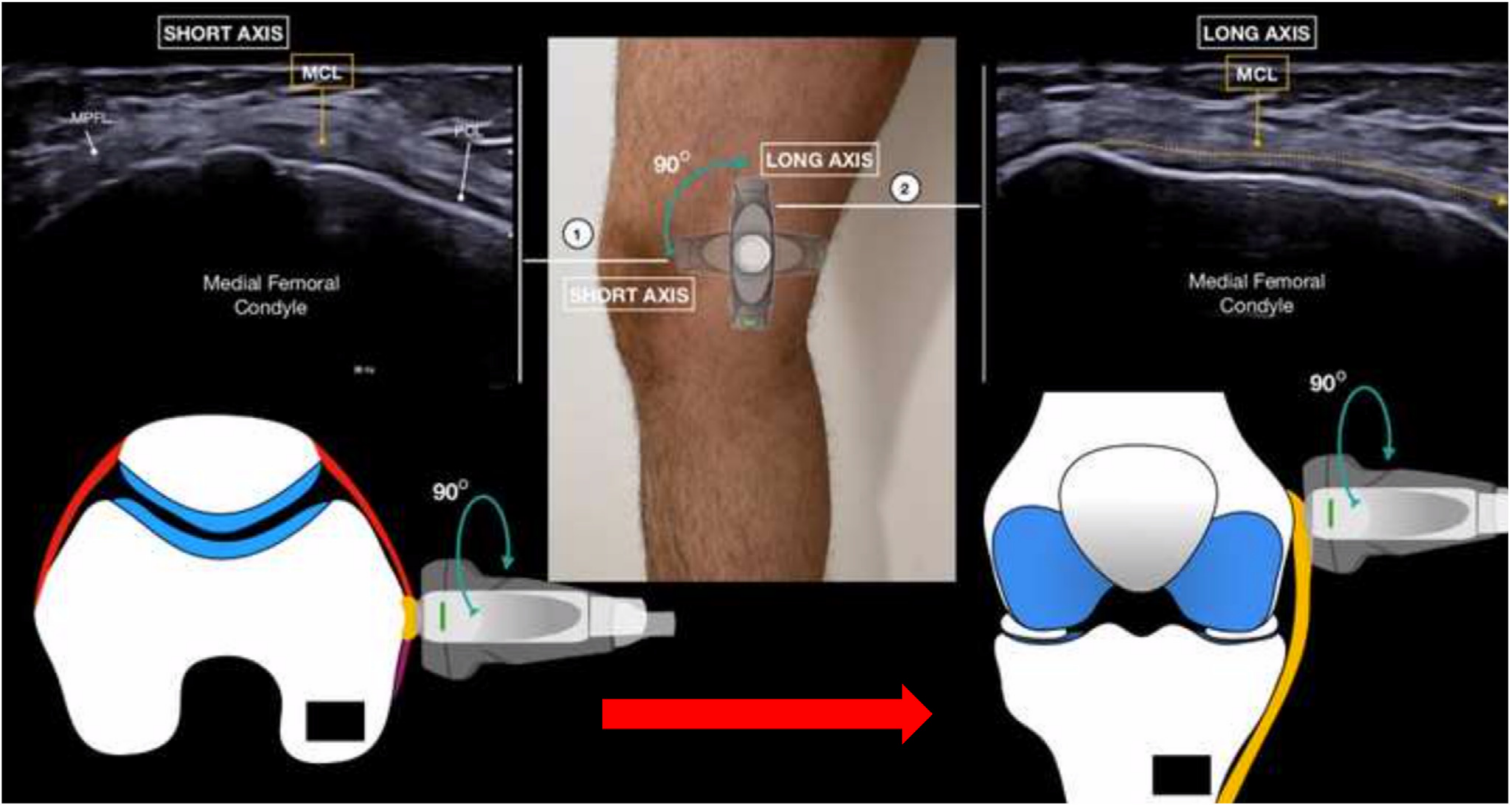
Once the MCL is identified on the ultrasound image, the probe should be held in place and rotated 90° such that it is in the long axis and follows the fibers of the MCL. (MCL, medial collateral ligament.)

**Fig 4:**
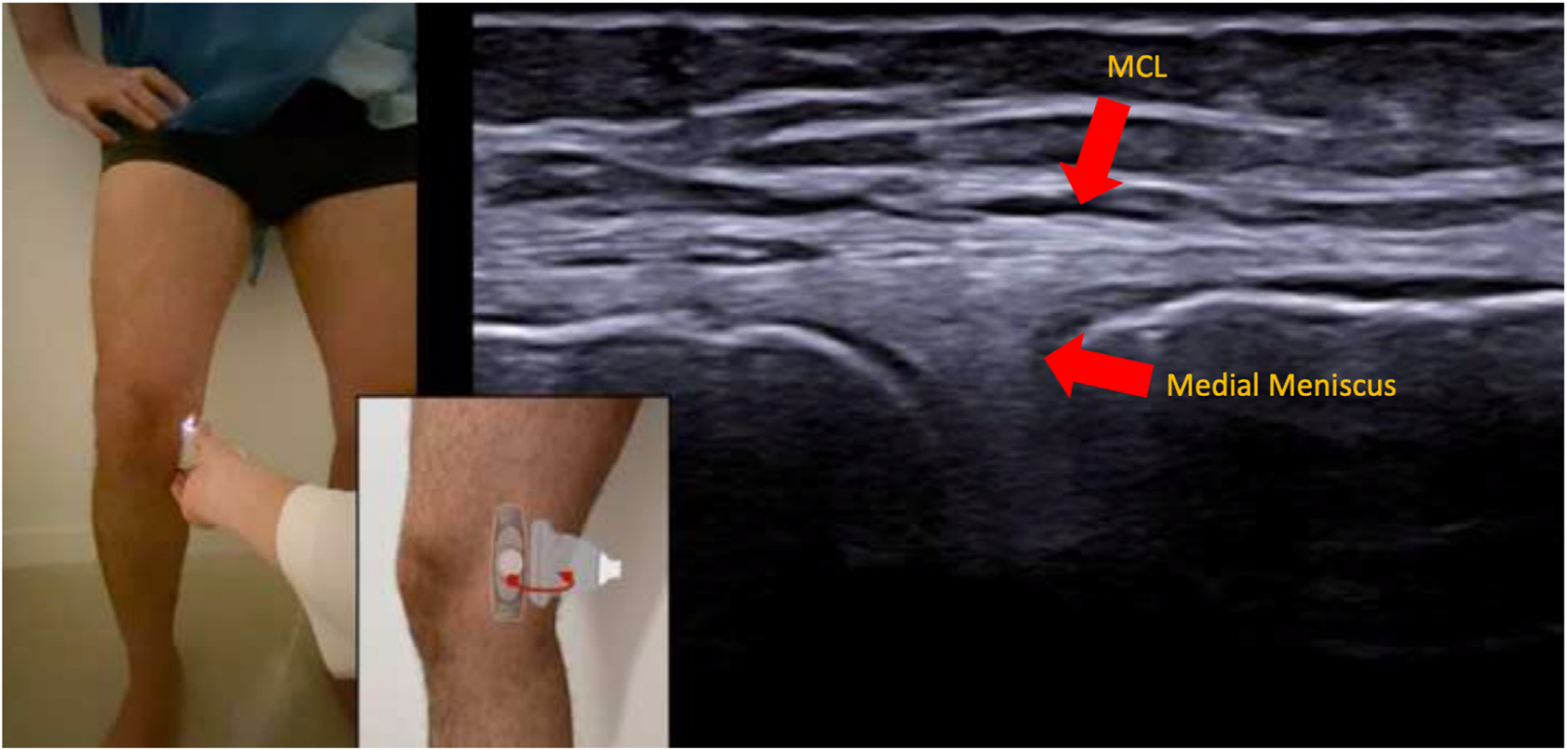
Once the probe is over the joint space on the ultrasound image, it should be shifted posteriorly such that it is over the posterior oblique ligament and the posterior border MPFL of the MCL. This is where ME will ultimately be measured. (MCL, medial collateral ligament; ME, meniscal extrusion; MPFL, medial patellofemoral ligament.)

During the procedure, the patient is standing freely such that the knee and menisci are experiencing physiologic axial loads. Ideally, if possible, the patient should bend the knees to approximately 30° of knee flexion (Fig 1A). If patients cannot tolerate this clinically, they can use a nearby wall or examination table for support via the upper extremities (Fig 1B and Video 1). Lastly, ME also can be measured in full extension (Fig 1C). Begin by palpating the medial femoral epicondyle and place the probe directly over in the short -axis plane. The first step is identifying the medial collateral ligament (MCL), which will be represented by an echogenic, circular image (Fig 2). The medial patellofemoral ligament can be found anteriorly, whereas the posterior oblique ligament can be found posteriorly. Once the MCL is identified, rotate the probe 90° such that the probe is now in the long axis and follows the fibers of the MCL (Fig 3). Slowly move the probe distally until the joint space and medial meniscus are visualized. Next, shift the probe posteriorly such that it is over the posterior oblique ligament and the posterior border of the MCL (Fig 4). This is where ME will ultimately be measured. At this coronal cross section, carefully move the probe around to achieve an image with the borders of the tibial cortex and medial meniscus best visualized. With the nonoperative hand or a helper, click the button on the machine to take a picture while the probe stays still, thus decreasing motion artifact. Before exporting the image, measure out a calibration line for future reference. Any distance is appropriate, but a line of 1 cm is a practical guide in this anatomic area.

**Fig 5:**
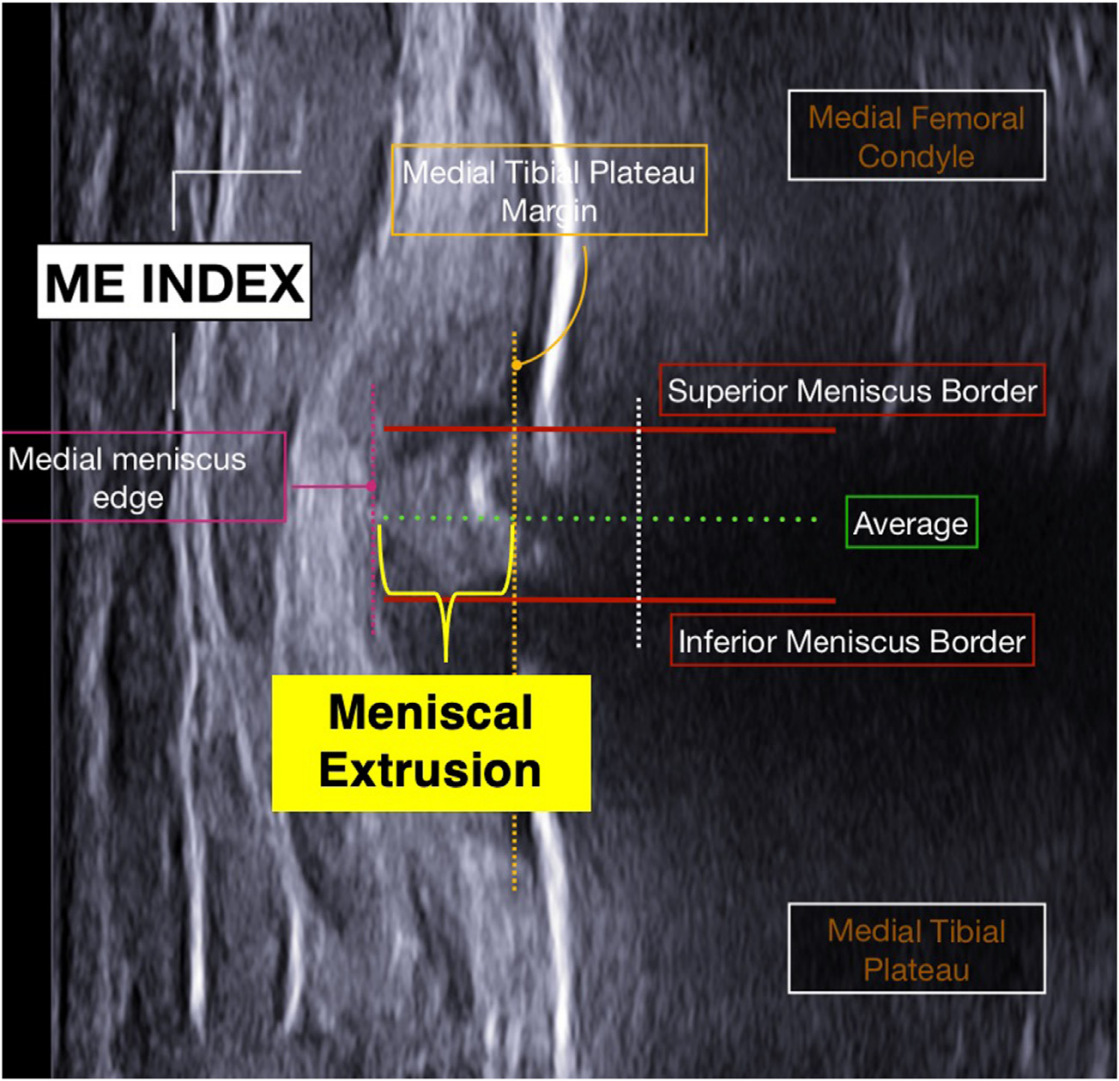
Once the probe is positioned over the posterior border of the MCL in the long-axis plane on the ultrasound image, we can begin making ME measurements. Draw a line parallel with the cortex of the proximal tibia at the articular margin. This will serve as a reference line and represent 0 millimeters of ME. Next, 2 lines at the superior and inferior meniscal borders should be drawn, with a third line going down the middle, denoted by “Average.” Lastly, a line should be drawn at the medial meniscal edge. The distance from the reference line and edge line will be the final meniscal extrusion measurement. (MCL, medial collateral ligament; ME, meniscal extrusion.)

Now that the image is ready, it can be annotated with our desired measurements. Begin by drawing out the reference line, which will represent 0 mm of ME (Fig 5). At the articular margin of the tibia, draw a line parallel with the cortex of the proximal tibia. In degenerative knees, osteophytes may be encountered. Careful attention should be made to exclude osteophytes as they can erroneously extend the medial edge of the tibia and inappropriately decrease the resultant measured ME. Once the reference line has been drawn, we will redirect our focus to the meniscus. Even in the 2-dimensional plane, the shape of the meniscus may vary, which in turn can affect ME measurements. Thus, a consistent area on the most superficial edge of the meniscus should be identified to make these measurements reproducible. The current authors believe measuring ME using the superior−inferior midpoint of the meniscal border would best allow for reproducible measurements. A second line from this point on the meniscus should be drawn such that it becomes perpendicular with the first reference line. This second line represents ME. If necessary, the calibration line can now be used to convert the distance of the second line from pixels into millimeters.

## Discussion

Meniscal displacement away from the tibial plateau is a 3-dimensional phenomenon, whereas ME is a 2-dimensional measurement, examining 1 specific cross section while not accounting for displacement elsewhere.^12^ This already calls into question the diagnostic utility of ME. Thus, it becomes even more important to ensure all operators use the same, standardized approach so variability is minimized. We believe the steps outlined in this technique article are practical and reproducible while not sacrificing accuracy (Table 1).

**Table 1:** Tips for Ultrasound Imaging of ME

Tips	Reasons
Place tension on skin	Move adipose tissue out of the way to better image joint line
Target posterior border of MCL at the joint line	Reproducible landmark that has been shown to be sensitive to ME
Ensure probe flat on skin	Maximize image resolution
Tilt probe left to right	Find cross section with best resolution of relevant anatomy
Use ultrasound software to create reference line	Create line converting pixels to millimeters if measurements to be made at later time

MCL, medial collateral ligament; ME, meniscal extrusion.

Patients are asked to stand during the examination such that axial loads can be applied to help displace an unstable meniscus off the tibial plateau. When possible, the current authors also recommend having patients in slight knee flexion since some studies have shown it to be the position that best facilitates ME in the setting of PMMRTs. When both the posterior medial meniscal root and the medial meniscotibial ligament were sectioned, our cadaveric analysis demonstrated ME to be significantly greater in 30° versus 0° of flexion.^13^ Using 3-dimensional digitization, Daney et al.^14^ similarly reported greater ME at greater degrees of knee flexion. We also recommend imaging the meniscus at the posterior border of the MCL for 2 main reasons. First, the MCL is a prominent structure that can be consistently visualized, regardless of operator experience, as a thick echogenic band on ultrasound. In other words, it offers clinicians a reproducible landmark. Second, there have already been several studies evaluating ME at the posterior border of the MCL, which have found greater levels of ME in this area compared with measuring at other coronal cross sections along the medial meniscus.^13^^,^^14^

With respect to measurement techniques, most available studies report using a reference line representing 0 mm of ME based on the location of only the medial tibial plateau, rather than a line connecting the vertices of the tibial and femoral cortices at the joint line.^11^^,^^15^ The latter approach is difficult to reproduce, given that the femoral condyle is convex; therefore, identifying the vertex where the joint line begins on the femur is challenging to identify. In turn, an accurate reference line becomes challenging to consistently create. Thus, we recommend only using the medial tibial plateau for drawing a reference line. As mentioned earlier, since the meniscus varies in shape, we recommend using the superior−inferior midpoint of the outermost edge of the meniscus to demarcate ME. It is important to point out because measuring ME from a slightly different part of the meniscus can substantially alter results.

The choice to measure ME with ultrasound certainly comes with both its benefits and drawbacks (Table 2). Although magnetic resonance imaging is considered the gold standard for measuring ME, ultrasound has been shown to yield comparable results. Nogueira-Barbosa et al.^16^ found good agreement when comparing ME measurement values between the 2 modalities. Our previous cadaveric analyses demonstrated a mean difference of only 0.25 mm when comparing ME values when measured with the 2 modalities.^13^^,^^17^ The desire to use ultrasound for ME stems from its practicality, ability for weight-bearing measurements, and affordability. There have been several studies that have even reported its utility for measuring ME after medial meniscus allograft transplantation and identifying patients with lateral discoid menisci.^18^, ^19^, ^20^ Although magnetic resonance images generally require the hassle of making appointments and time for processing, ultrasound images can immediately be done within the comfort of the examination room and provide instant results. Next, the ability for weight-bearing measurements allows for subtler pathologies to be better highlighted, such that they can be caught when imaged. Of course, however, the quality of imaging with ultrasound is only as good as the operator. Thus, a baseline level of experience is generally necessary to achieve consistent results. In summary, we describe a technique for measuring medial ME with ultrasound that is technically simple and very reproducible.

**Table 2:** Advantages and Disadvantages of Measuring Meniscal Extrusion With Ultrasound Imaging

Advantages	Disadvantages
Easily accessible in clinic	Operator dependent
Allows for dynamic measurements	Only captures one cross-sectional image at a time
Affordable	Lower-resolution image compared with MRI
	Anatomical landmarks not always clear

MRI, magnetic resonance imaging.

## Disclosures

The authors declare the following financial interests/personal relationships which may be considered as potential competing interests: M.H. reports a relationship with DJO-Enovis that includes: consulting or advisory; a relationship with Elsevier that includes: equity or stocks; a relationship with *Journal of Cartilage and Joint Preservation* that includes: board membership; a relationship with Moximed that includes: consulting or advisory; and a relationship with Vericel Corporation that includes: consulting or advisory. J.C. reports a relationship with 10.13039/100011549American Orthopaedic Society for Sports Medicine that includes: board membership, a relationship with 10.13039/100007307Arthrex Inc that includes: consulting or advisory; a relationship with 10.13039/100008542Arthroscopy Association of North America that includes: board membership; a relationship with CONMED Linvatec that includes: consulting or advisory; a relationship with International Society of Arthroscopy Knee Surgery and Orthopaedic Sports Medicine that includes: board membership; a relationship with Ossur that includes: consulting or advisory; and a relationship with Smith & Nephew Inc. that includes: speaking and lecture fees. All other authors (D.F., T.A.P.) declare that they have no known competing financial interests or personal relationships that could have appeared to influence the work reported in this paper.

## Supplementary Data

Video 1 Detailed steps of the technique with a demonstration is described with a voiceover. The video shows the patient first placed in 30° of knee flexion while weight-bearing. The image is captured at the posterior border of the medical collateral ligament—a reproducible landmark on ultrasound marked by both palpation and a dense echogenicity on imaging. A reference line parallel to the articular margin of the tibia is then drawn to represent 0 millimeters of meniscal extrusion, and a perpendicular line is drawn from there to the outermost edge of the meniscus at its coronal midpoint to arrive at the final meniscal extrusion value.

## References

[1] Allaire R., Muriuki M., Gilbertson L., Harner C.D. (2008). Biomechanical consequences of a tear of the posterior root of the medial meniscus: Similar to total meniscectomy. J Bone Joint Surg Am.

[2] Englund M., Guermazi A., Gale D. (2008). Incidental meniscal findings on knee MRI in middle-aged and elderly persons. N Engl J Med.

[3] Costa C.R., Morrison W.B., Carrino J.A. (2004). Medial meniscus extrusion on knee MRI: Is extent associated with severity of degeneration or type of tear?. AJR Am J Roentgenol.

[4] Crema M.D., Roemer F.W., Felson D.T. (2012). Factors associated with meniscal extrusion in knees with or at risk for osteoarthritis: The Multicenter Osteoarthritis Study. Radiology.

[5] DePhillipo N.N., Kennedy M.I., Chahla J., LaPrade R.F. (2019). Type II medial meniscus root repair with peripheral release for addressing meniscal extrusion. Arthrosc Tech.

[6] Lerer D.B., Umans H.R., Hu M.X., Jones M.H. (2004). The role of meniscal root pathology and radial meniscal tear in medial meniscal extrusion. Skeletal Radiol.

[7] Liu Y., Joseph G.B., Foreman S.C. (2021). Determining a threshold of medial meniscal extrusion for prediction of knee pain and cartilage damage progression over 4 years: Data from the osteoarthritis initiative. AJR Am J Roentgenol.

[8] Muzaffar N., Kirmani O., Ahsan M., Ahmad S. (2015). Meniscal extrusion in the knee: Should only 3 mm extrusion be considered significant? An assessment by MRI and arthroscopy. Malays Orthop J.

[9] Kawaguchi K., Enokida M., Otsuki R., Teshima R. (2012). Ultrasonographic evaluation of medial radial displacement of the medial meniscus in knee osteoarthritis. Arthritis Rheum.

[10] Svensson F., Felson D.T., Turkiewicz A. (2019). Scrutinizing the cut-off for ‘‘pathological’’ meniscal body extrusion on knee MRI. Eur Radiol.

[11] Farivar D., Hevesi M., Fortier L.M., Azua E., LaPrade R.F., Chahla J. (2023). Meniscal extrusion measurements after posterior medial meniscus root tears: A systematic review and meta-analysis. Am J Sports Med.

[12] Emmanuel K., Quinn E., Niu J. (2016). Quantitative measures of meniscus extrusion predict incident radiographic knee osteoarthritis: Data from the Osteoarthritis Initiative. Osteoarthritis Cartilage.

[13] Farivar D., Knapik D.M., Vadhera A.S. (2023). Medial meniscal extrusion of greater than 3 millimeters on ultrasound suggests combined medial meniscotibial ligament and posterior medial meniscal root tears: A cadaveric analysis. Arthroscopy.

[14] Daney B.T., Aman Z.S., Krob J.J. (2019). Utilization of transtibial centralization suture best minimizes extrusion and restores tibiofemoral contact mechanics for anatomic medial meniscal root repairs in a cadaveric model. Am J Sports Med.

[15] Boxheimer L., Lutz A.M., Treiber K. (2004). MR imaging of the knee: Position related changes of the menisci in asymptomatic volunteers. Invest Radiol.

[16] Nogueira-Barbosa M.H., Gregio-Junior E., Lorenzato M.M. (2015). Ultrasound assessment of medial meniscal extrusion: A validation study using MRI as reference standard. AJR Am J Roentgenol.

[17] Farivar D., Knapik D.M., Vadhera A.S. (2023). Isolated posterior lateral meniscofemoral ligament tears show greater meniscal extrusion in knee extension, and isolated posterior lateral meniscal root tears show greater meniscal extrusion at 30° using ultrasound: A cadaveric study. Arthroscopy.

[18] Condron N.B., Knapik D.M., Gilat R. (2022). Concomitant meniscotibial ligament reconstruction decreases meniscal extrusion following medial meniscus allograft transplantation: A cadaveric analysis. Arthroscopy.

[19] Yang S.J., Zhang M.Z., Li J., Xue Y., Chen G. (2021). A reliable, ultrasound-based method for the diagnosis of discoid lateral meniscus. Arthroscopy.

[20] Everhart J.S., Tysklind R.G. (2021). Editorial Commentary: The pediatric knee: Ultrasound could replace magnetic resonance imaging for evaluating a discoid lateral meniscus. Arthroscopy.

